# Detection and characterization of a MERS-related coronavirus (*Betacoronavirus cameli*) in velvety free-tailed bats (*Molossus molossus*, Mammalia) captured in Southern Brazil

**DOI:** 10.1007/s42770-026-02034-3

**Published:** 2026-07-29

**Authors:** Alexandre Sita, Micheli Filippi, Juliana Comerlato, Gabriela Espindola Birlem, Deivid de Souza da Silva, Larissa Mallmann, Vyctoria Malahyka de Abreu Góes Pereira, Meriane Demoliner, Juliana Schons Gularte, Alana Witt Hansen, Fernando Rosado Spilki, Matheus Nunes Weber

**Affiliations:** 1 Laboratório de Microbiologia Molecular, Universidade Feevale, Novo Hamburgo, RS Brazil; 2 Laboratório de Imunologia e Biologia Molecular, Faculdade de Veterinária, Universidade Federal do Rio Grande do Sul (UFRGS), Porto Alegre, RS Brazil

**Keywords:** Merbecovirus, Chiropterans, One Health, Betacoronavirus, Eco vigilance

## Abstract

Bat-borne coronaviruses are of increasing interest due to their potential for zoonotic spillover, particularly within the *Betacoronavirus* genus. Partial genomic sequences related to MERS-like CoV was identified in multiple hosts, including humans, camelids, and bats from the families Vespertilionidae and Molossidae. In this study, we investigated the presence of coronaviruses in 65 *Molossus molossus* bats captured in southern Brazil using nested RT-PCR and high-throughput sequencing (HTS). One individual (1.5%) tested positive in a rectal swab, and sequence analysis revealed the presence of a MERS-related coronavirus (*Betacoronavirus cameli*). Phylogenetic reconstruction based on five genomic contigs clustered the virus within the *Merbecovirus* subgenus, confirming its close relationship to MERS-CoV strains previously detected in bats and dromedary camels. *M. molossus* is a widely distributed, insectivorous bat species with synanthropic behavior, commonly roosting in urban areas and human dwellings. The detection of a MERSr-CoV in this species in southern Brazil expands the known host range of these viruses and suggests that members of the Molossidae family may serve as reservoirs. These findings underscore the importance of continuous genomic surveillance in Neotropical bats, particularly in the context of environmental changes that intensify contact between wildlife, humans, and domestic animals.

**Supplementary Information:**

The online version contains supplementary material available at 10.1007/s42770-026-02034-3.

## Introduction

Anthropogenic changes are accelerating environmental degradation and increasing human exposure to various bat species and their associated microbiota. Such interactions heighten the risk of exposure to emerging pathogens, and the growing proximity between wild and domestic animals exacerbates this risk [1, 2]. Bats serve as natural reservoirs for several important zoonotic viruses, including rabies virus, Hendra and Nipah viruses, and a wide diversity of coronaviruses (CoVs) [2–5]. Many bat species are highly gregarious, often forming dense colonies and sharing roosting sites with diverse assemblages of other bat species [2, 6]. In addition, their distinct immune system, characterized by a dampened inflammatory response, may make bats particularly well-suited to harbor and potentially transmit zoonotic viruses to humans [7].

The genus *Betacoronavirus* (β-CoV) includes viruses responsible for severe respiratory diseases in humans, such as severe acute respiratory syndrome coronavirus (SARS-CoV) and SARS-CoV-2 (both classified within the subgenus *Sarbecovirus*) as well as Middle East respiratory syndrome coronavirus (MERS-CoV), which belongs to the subgenus *Merbecovirus* [8]. These viruses have raised significant concern due to their zoonotic potential and their presumed origins in bats [9–11]. Moreover, bats are recognized as important reservoirs and potential intermediate hosts in the transmission dynamics of several β-CoVs [11, 12].

MERS-CoV was first identified in 2012 and has since emerged as a significant zoonotic pathogen, causing recurrent outbreaks in human populations in Saudi Arabia and exhibiting a case fatality rate of approximately 36% [13, 14]. According to the International Committee on Taxonomy of Viruses (ICTV), the *Merbecovirus* subgenus comprises four distinct species: *Betacoronavirus cameli* (MERS-related CoV, MERSr-CoV), *Betacoronavirus erinacei* (Erinaceus coronavirus, EriCoV), *Betacoronavirus pipistrelli* (HKU5), and *Betacoronavirus tylonycteridis* (HKU4) [8]. Partial MERS-related CoV genomic sequences were identified in multiple hosts, including humans, camelids, and bats from the families Vespertilionidae (*Neoromicia capensis*, *Eptesicus serotinus*, *Pipistrellus kuhlii*, *Hypsugo savii*, *Vespertilio sinensis*, and *Myotis ricketti*) from China and South Africa [15, 16], and Molossidae (*Eumops glaucinus* and *Molossus molossus*) from Brazil [17, 18].

The circulation of coronaviruses in tropical and subtropical bat populations highlights a relevant One Health concern, particularly in Brazil, where biodiversity, synanthropic wildlife species, and increasing anthropogenic pressures converge to create hotspots for zoonotic emergence [5]. The velvety free-tailed bat (*Molossus molossus*) is a widespread insectivorous species of chiropteran belonging to the family Molossidae. It inhabits tropical and temperate regions of Central and South America, primarily occupying open forests and urban areas, and roosting in caves, house attics, and abandoned buildings [19]. The velvety free-tailed bat is not only widely distributed across the Neotropics but also frequently roosts in human dwellings, enhancing the potential for viral spillover. Monitoring the health of this bat species is important, as previous studies have reported the presence of rabies virus [20], alphacoronaviruses (α-CoVs) [21], and β-CoVs [18] in *M. molossus*. In this study, we investigated the presence of coronaviruses in *M. molossus* and report the detection of a MERSr*-*CoV in a specimen from southern Brazil.

## Materials and methods

### Samples

**Fig. 1:**
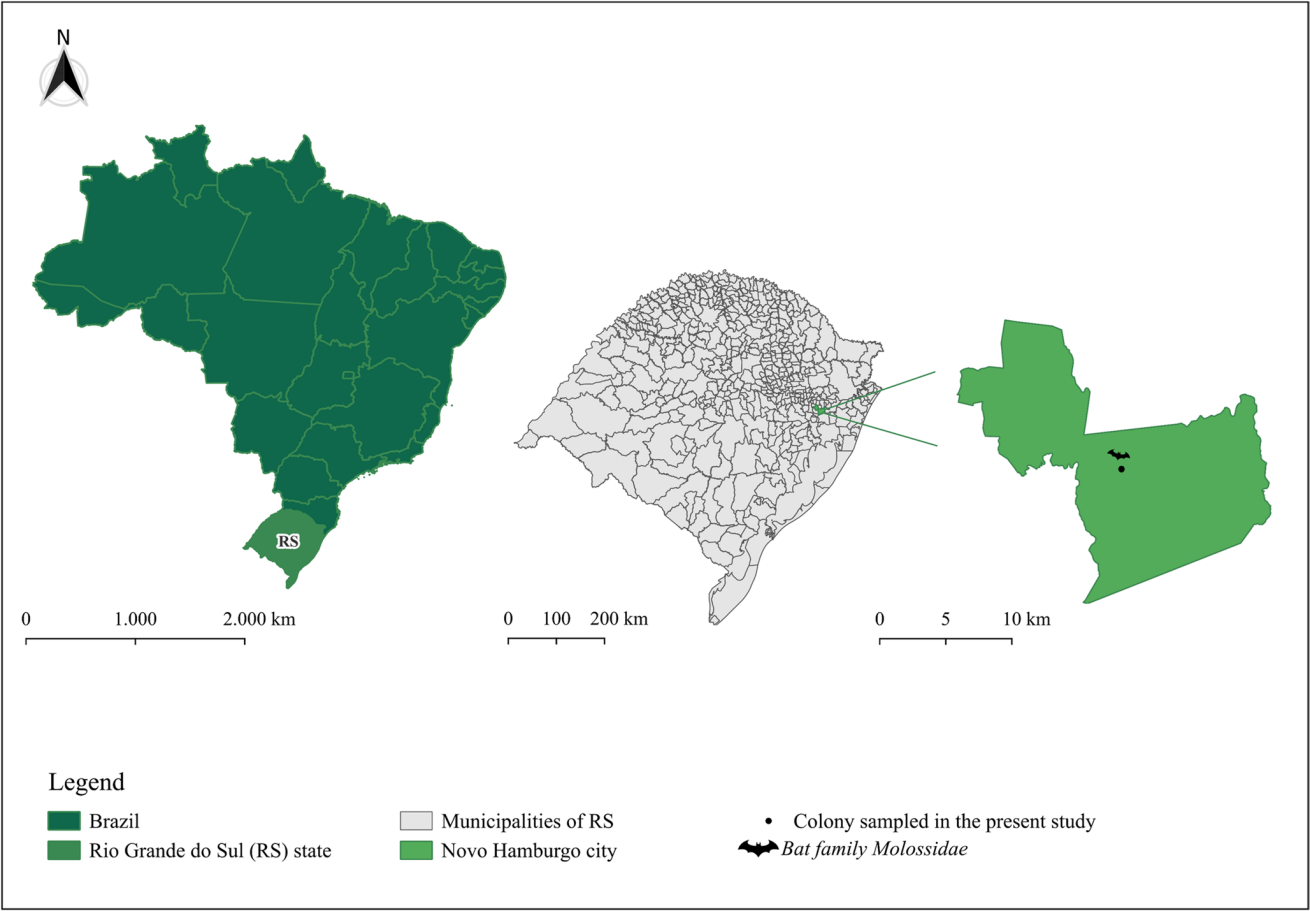
Geographical distribution of bat capture sites analyzed in the present study

Oral and rectal swabs were collected from 65 *Molossus molossus* bats between March and December 2022. The bats were captured using mist net in a rural area of the municipality of Novo Hamburgo, in the state of Rio Grande do Sul, southern Brazil (29°44’19.7"S, 51°03’03.7"W) (Fig. 1). The bats collected were roosting in the attic of an abandoned house at a density exceeding 100 individuals. The taxonomic identification of *Molossus molossus* was based on external and cranial morphological characteristics, including forearm length, ear morphology, tail extension beyond the uropatagium, and dental features, according to the criteria established for the family Molossidae and the genus *Molossus* [22, 23].

The animals were physically restrained only; they were not anesthetized or euthanized. During handling, oral and rectal swabs (Sterile nylon flocked nasal swab, Global Trade Technology, Jaboticabal, Brazil) were collected, the bats were photographed, and then promptly released at the capture site. Swabs from each individual were stored separately in tubes containing 1 mL of RNAlater™ Stabilization Solution (Thermo Fisher Scientific™, Waltham, MA, USA), transported in an insulated container, and stored at − 80 °C until further processing.

This study was approved by the Ethics Committee on Animal Use of Universidade Feevale (protocol number 2.21.097) and authorized by the Chico Mendes Institute for Biodiversity Conservation (ICMBio) (protocol number 78499–1).

### Nucleic acids isolation and PCR

RNA and DNA were isolated using the MagMAX™ CORE Nucleic Acid Purification Kit (Thermo Fisher Scientific™, Waltham, MA, USA) on the automated KingFisher™ Duo Prime system (Thermo Fisher Scientific™), following the manufacturer’s instructions. Complementary DNA (cDNA) was synthesized using the GoScript™ Reverse Transcriptase Kit with Random Primers (Promega Corporation, Madison, WI, USA), according to the manufacturer’s protocol. PCRs were performed using a pan-CoV primer set in order to amplify a segment of RNA-dependent RNA polymerase (RdRp) in ORFab [24] and GoTaq^®^ Colorless Master Mix (Promega Corporation), following the manufacturer’s guidelines. First round and nested PCR reactions were conducted using the following cycle conditions: initial denaturation of 95^o^ C during 5 min; 35 cycles of 95o C during 45 s, 50^o^ C during 45 s and 72^o^ C during 45 s; final extension of 72^o^ C during 7 min. PCR products were stained with ethidium bromide, separated by electrophoresis on a 2% agarose gel, and visualized under ultraviolet light.

### Purification, sequencing, and phylogenetic analysis

PCR-positive products were purified using the PureLink™ PCR Purification Kit (Thermo Fisher Scientific™, Waltham, MA, USA), following the manufacturer’s instructions. Bidirectional sequencing was performed at ACTGene Laboratory (Alvorada, RS, Brazil) using an ABI PRISM^®^ 3100 Genetic Analyzer equipped with 50 cm capillaries and POP-6 polymer (Applied Biosystems, Waltham, MA, USA).

### Pan-coronavirus panel sequencing

Samples that tested negative for coronavirus by PCR were grouped into four pools of 32 individuals each, according to the anatomical sampling site (oral or rectal). The sample that tested positive was analyzed individually in two separate pools (oral and rectal). Total nucleic acids were isolated using the MagMAX™ CORE Nucleic Acid Purification Kit (Thermo Fisher Scientific™, Waltham, MA, USA) on the automated KingFisher™ Duo Prime System (Thermo Fisher Scientific™), in a final volume of 200 µL and eluted in 90 µL, following the manufacturer’s instructions.

For RNA reverse transcription and library preparation, the Illumina^®^ RNA Prep with Enrichment (L) Tagmentation kit (Illumina^®^, San Diego, CA, USA) was used in combination with oligonucleotides from the Pan-Coronavirus Panel (Illumina^®^), according to the manufacturer’s protocol. Sample quantity and quality were assessed by fluorometry and automated electrophoresis using a Qubit™ Fluorometer (Thermo Fisher Scientific™) and a TapeStation 4150 System (Agilent Technologies, Santa Clara, CA, USA), respectively. Sequencing was performed on the NextSeq 1000 platform (Illumina^®^) using a NextSeq 1000/2000 P1 Reagent Cartridge (300 cycles).

### Bioinformatic analysis

Nucleotide sequences were edited using the Geneious Prime 2022.1 bioinformatics suite (https://www.geneious.com). The Chan Zuckerberg ID platform (Chan Zuckerberg Initiative, https://czid.org) and Genome Detective (https://www.genomedetective.com) were used for sequence assembly and metagenomic analysis. Samples were compared with representative sequences available in the GenBank database using the nucleotide BLAST tool (https://blast.ncbi.nlm.nih.gov/Blast.cgi).

Sequences were aligned using the MAFFT program [25]. Phylogenetic analyses based on five genomic contigs were conducted individually and concatenated. The concatenation of the sequences was performed using Concatenator version 0.3.1 software [26]. For evaluation of the concatenation, the Incongruence-length-difference (ILD) test was done using WinClada software [27].

The phylogenetic reconstructions were conducted using Maximum Likelihood (ML) inference with the GTR + F+I+G4 substitution model implemented in IQ-TREE software [28]. The best-fit substitution model was selected for each hypothesis using the ModelFinder tool. The robustness of the phylogenetic relationships was assessed by performing 1,000 bootstrap replicates.

## Results

In the present study, 65 *M. molossus* individuals were tested by nested RT-PCR using a pan-coronavirus primer set [24] on oral and rectal swabs. One out of 65 (1.54%) rectal swab samples tested positive, while all oral swabs tested negative. The positive individual was an adult female that appeared clinically healthy. The PCR-positive sample was submitted for dideoxy sequencing and, according to BLASTn analysis, showed 99.2% nucleotide identity with a MERSr-CoV previously detected in an *Eumops glaucinus* molossid bat in southeastern Brazil in 2013 (GenBank accession number KT717386.1). This sequence also presented 99% of nucleotide identity with a MERSr-CoV previously detected in *Molossus molossus* bat in northheastern Brazil in 2023 (GenBank accession number SRR30934467).

**Fig. 2:**
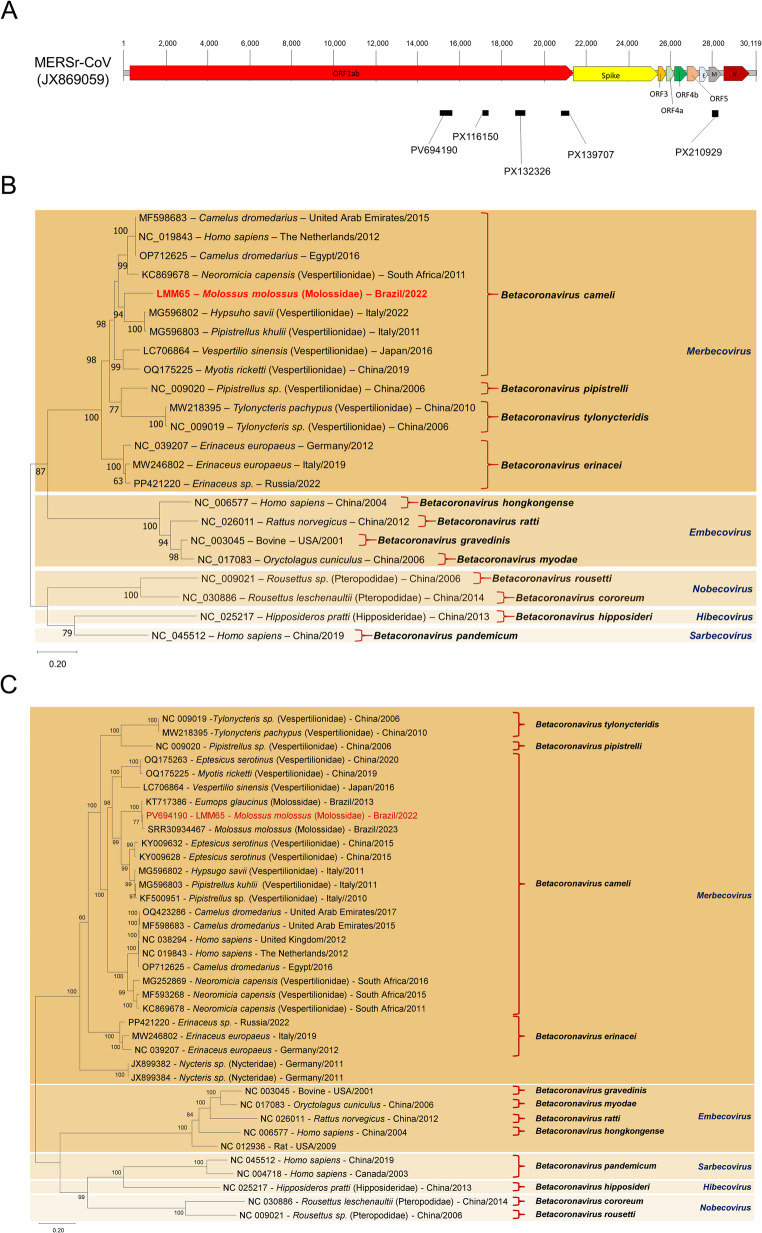
The genetic characterization of betacoronavirus observed in the present study. **A** Genome position of MERSr-CoV sequences observed in the present study compared with a prototype. **B** Nucleotide phylogenetic reconstruction of ORF1ab partial sequences constructed using IQ-TREE software using maximum likelihood inference the GTR + F+I+G4 model in 1,000 bootstrap replicates. **C** Concatenated nucleotide phylogenetic reconstruction of the five genomic sequences (four into ORF1ab gene and one in membrane protein) constructed using IQ-TREE software using maximum likelihood inference the GTR + F+I+G4 model in 1,000 bootstrap replicates

Samples that tested negative for coronavirus by PCR [24] were pooled and submitted for high-throughput sequencing (HTS) using a commercial pan-coronavirus panel, while the positive sample [24] was analyzed individually. The 64 individuals that tested negative by nested RT-PCR [24] showed no coronavirus sequence reads. The PCR-positive sample generated 1,093,091 reads and yielded four contigs (sizes: 245, 437, 402, and 246 nt) corresponding to the ORF1ab gene and membrane (M) protein (BioSample SAMN61430959) (Fig. 2A). These four contigs were generated by 12, 18, 30, and 8 reads in HTS, respectively. The three partial ORF1ab contigs showed nucleotide identities of 88.9%, 86.4%, and 82.5% with MERSr-CoVs previously reported *Camelus dromedaries* from United Arab Emirates in 2016, *Camelus dromedarius* from Saudi Arabia in 2023, and *Myotis ricketti* bat from China in 2019 (GenBank accession numbers OQ423283.1, PP952197.1, and OQ175225.1, respectively). The M protein sequence presented 83.5% of nucleotide identity with a MERSr-CoV reported in a *Neoromicia capensis* bat in South Africa in 2015 (GenBank accession number MF593268.1).

In phylogenetic reconstructions, the sequences obtained in the present study (Fig. 2A) clustered within the *Betacoronavirus cameli* clade in all hypothesis. In the phylogenetic reconstruction of partial RNA polymerase RNA dependent sequences, LMM 65 clustered in the same node with MERSr-CoVs reported in the molossid bats *Molossus molossus* (GenBank accession number SRR30934467) and *Eumops glaucinus* (GenBank accession number KT717386) supported by a 100% bootstrap value (Fig. 2B). In the four contigs generated from the pan-coronavirus panel in HTS, our sequences also clustered within the *B. cameli* clade, further reinforcing the robustness of our findings. In ORF1ab segments (Figures S1, S2 and S3), LMM clustered in the same terminal node of MERSr-CoVs reported in *Myotis ricketti* and *Vespertilio sinensis* bats (GenBank accession numbers OQ175225 and LC706864, respectively) (Figure S1 and S3), MERSr-CoVs reported in humans, camelids and vespertilionid bats (Figure S2). In the M gene segment, LMM 65 clustered in an individual branch into *B. cameli* clade (Figure S4).

The ILD test demonstrated that the five contigs datasets (Fig. 2A) were not significantly incongruent (*P* = 0.1997). Therefore, an approach combining the five sequences obtained of LMM 65 was selected to reveal the relationship between our sample and other betacoronaviruses (β-CoVs). In the concatenated phylogenetic tree (Fig. 2C), LMM 65 clustered within the *B. cameli* clade in the same terminal node with MERSr-CoVs reported in *Hypsugo savii* and *Pipistrellus kuhlii* bats from Italy (GenBank accession numbers MG596802 and MG596803). It was not possible to insert other MERSr-CoV sequences detected in Brazil since they were only partial genomes and not homologous to our sequences into the concatenated approach.

## Discussion

In the present study, we analyzed the presence of coronavirus in oral and rectal swabs from 65 *Molossus molossus* bats captured in southern Brazil using a combination of PCR and HTS (Fig. 1). One individual (1.5%) tested positive in a rectal swab and was classified as a betacoronavirus (β-CoV) in all phylogenetic reconstructions applied, including individual phylogenetic reconstructions of ORF1ab (Figs. 2B, S1, S2, and 3) and M (Figure S4) segments, and a concatenated ML analysis (Fig. 2C). Most coronavirus detections in bats in South America have reported the presence of alphacoronaviruses (α-CoVs) [5, 17, 29–31]. Reports of β-CoVs in South America are scarce [5, 17, 18, 32], but genomic surveillance of this group of CoVs in bats is important, as chiropterans may serve as bridge hosts for β-CoVs associated with severe human infections [4, 12]. Recently, a new β-CoV putative subgenus proposed as *Ambecovirus* (American β-CoV) was reported in bats in Northeastern Brazil [33], but these sequences appear to be different from those reported in the present study that grouped in *Merbecovirus* clade (Fig. 2B and C).

Chiropterans are potential hosts of merbecovirus, including bats from the family Nycterdae from Ghana and Guinea [34, 35], Emballonuridae from Saudi Arabia and Zimbabwe [33, 36], Molossidae from Mexico [34], and Mormoopidae from Mexico [35]. MERS-related coronaviruses (MERSr-CoVs) have been reported in vespertilionid bats (*Neoromicia capensis*, *Eptesicus serotinus*, *Pipistrellus kuhlii*, *Hypsugo savii*, *Vespertilio sinensis*, and *Myotis ricketti*) from China and South Africa [15, 16], as well as in *Molossus molossus Eumops glaucinus* molossid bats in northeastern [18] and southeastern Brazil [17]. In the present study, we detected a MERSr-CoV in a *Molossus molossus* bat from southern Brazil (Fig. 2B and C) which reinforce the expansion of the known host range of merbecoviruses within Molossidae.

Brazil’s large territorial extent and ecological heterogeneity may favor the diversification of bat-associated merbecoviruses [4, 12], and the main bat families present in Brazil, such as Phyllostomidae, Molossidae, Emballonuridae, Vespertilionidae, Thyropteridae, Mormoopidae, Natalidae, and Noctilionidae, have broad geographic distributions encompassing various biomes, including the Amazon, Cerrado, Caatinga, Pantanal, and Atlantic Forest [19].

CoV demarcation criteria is based upon viruses for which a complete genome sequence is available or else a nearly complete genome that includes the 3CLpro, NiRAN, RdRP, ZBD and HEL1 domains. Demarcation thresholds are set as a range of percentage of unchanged differences (PUD) values, which are deduced from pairwise patristic distances (PPD) values for which the number of taxa clusters remained constant (https://ictv.global/report/chapter/coronaviridae/coronaviridae) [8]. It is important to highlight that the majority of works that reported MERSr-CoV in bats [15–17] only provided partial sequences of RdRp gene, which make difficult to correct classify them as *B. cameli*. MERS-related coronaviruses (MERSr-CoVs; *Betacoronavirus cameli*) have been identified in dromedary camels (*Camelus dromedarius*), which serve as the primary hosts associated with zoonotic transmission to humans [13, 14]. Phylogenetic evidence supports bats as ancestral hosts of MERS-related viruses [10, 13, 15]. Our findings in *Molossus molossus* reinforce the expansion of the host range of MERSr-CoV, consistent with those reported by Silvério et al. [18] in *Molossus molossus* and Goés et al. [17] in *Eumops glaucinus*,reinforcing the role of Molossidae as reservoirs of merbecoviruses in South American territory. Moreover, apparently the molossid bats harbor similar MERSr-CoV grouped in a same branch in phylogenetic tree (Fig. 2B). These findings emphasize that spatially separated populations can harbor genetically related merbecoviruses (Fig. 2B) highlighting the need for focused surveillance of high-pathogenicity coronaviruses in bats.

Despite the combined use of PCR and HTS in our study, the probable low quantity of CoV RNA limited the sequencing of longer genomic segments (Fig. 2A). Nevertheless, our findings provide evidence of a MERS-related β-CoV in South American molossid bats. *Molossus molossus* are insectivorous bats with synanthropic behavior, frequently roosting on rooftops and in abandoned buildings [19]. This close contact with humans increases opportunities for viral exposure ate the human-wildlife interface.

The study is limited by the partial genomic dataset further restricts phylogenetic resolution and recombination analyses, as well as comparisons of functionally important regions, such as the spike protein. In addition, the pooling strategy used for HTS may have obscured low-abundance viruses, leading to an underestimation of viral diversity. Finally, sampling from a single location limits broader inference about the geographic distribution and variability of *M. molossus* populations.

Thus, our findings contribute to a better understanding of the viral diversity of β-CoVs and underscore the importance of ecological surveillance through targeted investigations in bats, given their role as reservoirs of diverse viruses with potential for spillover and zoonotic events. Moreover, our work reinforces the expansion of the known host range of MERSr-CoVs and suggest bats of family Molossidae as potential hosts of this β-CoVs species. Anthropogenic changes are accelerating environmental degradation and increasing human and domestic animal exposure to wildlife microbiomes. This increased contact elevates the risk of transmissible infections with significant pathogenic potential, emphasizing the need for integrated approaches within the One Health framework.

## Supplementary Information

Below is the link to the electronic supplementary material.

Supplementary figure 1 (PNG 588 KB)

Supplementary Material 1 Figure S1. Nucleotide phylogenetic reconstruction of ORF1ab partial sequences constructed using IQ-TREE software using maximum likelihood inference the GTR+F+I+G4 model in 1,000 bootstrap replicates.
High Resolution Image (TIF 1.61 MB)

Supplementary figure 2 (PNG 632 KB)

Supplementary Material 2 Figure S2. Nucleotide phylogenetic reconstruction of ORF1ab partial sequences constructed using IQ-TREE software using maximum likelihood inference the GTR+F+I+G4 model in 1,000 bootstrap replicates.
High Resolution Image (TIF 1.70 MB)

Supplementary figure 3 (PNG 588 KB)

Supplementary Material 3 Figure S3. Nucleotide phylogenetic reconstruction of ORF1ab partial sequences constructed using IQ-TREE software using maximum likelihood inference the GTR+F+I+G4 model in 1,000 bootstrap replicates.
High Resolution Image (TIF 1.61 MB)

Supplementary figure 4 (PNG 564 KB)

Supplementary Material 4 Figure S4. Nucleotide phylogenetic reconstruction of membrane (M) protein partial sequences constructed using IQ-TREE software using maximum likelihood inference the GTR+F+I+G4 model in 1,000 bootstrap replicates.
High Resolution Image (TIF 1.54 MB)
