# Supplementary figures and images for: Detection and characterization of a MERS-related coronavirus (Betacoronavirus cameli) in velvety free-tailed bats (Molossus molossus, Mammalia) captured in Southern Brazil
Source: Braz J Microbiol. 2026 Jul 29;57(1):224. doi: 10.1007/s42770-026-02034-3 (PMC13421570; doi:10.1007/s42770-026-02034-3)

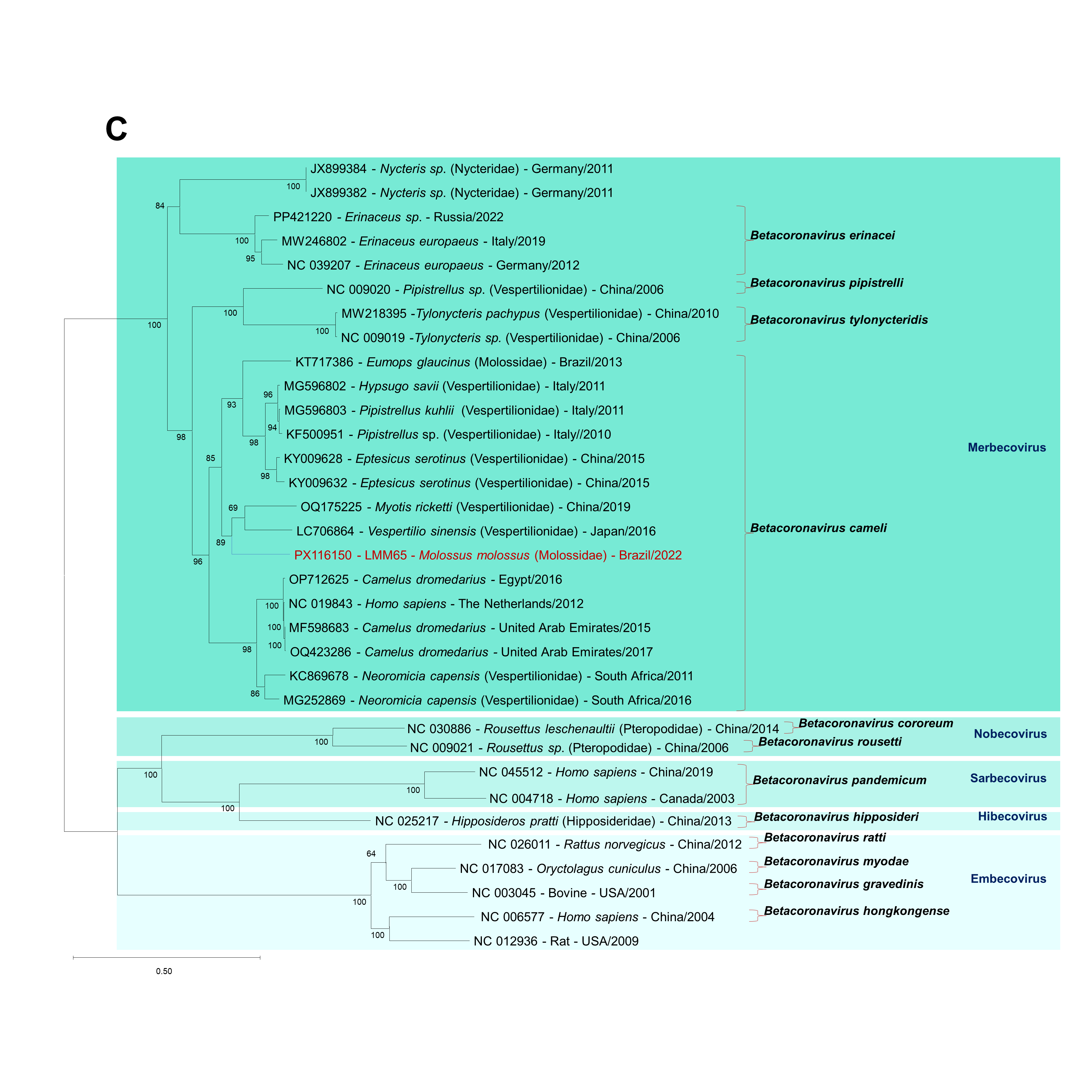

Supplement: Supplementary file 1 — Supplementary figure 1 (PNG 588 KB) [file 42770_2026_2034_Fig3_ESM.png]

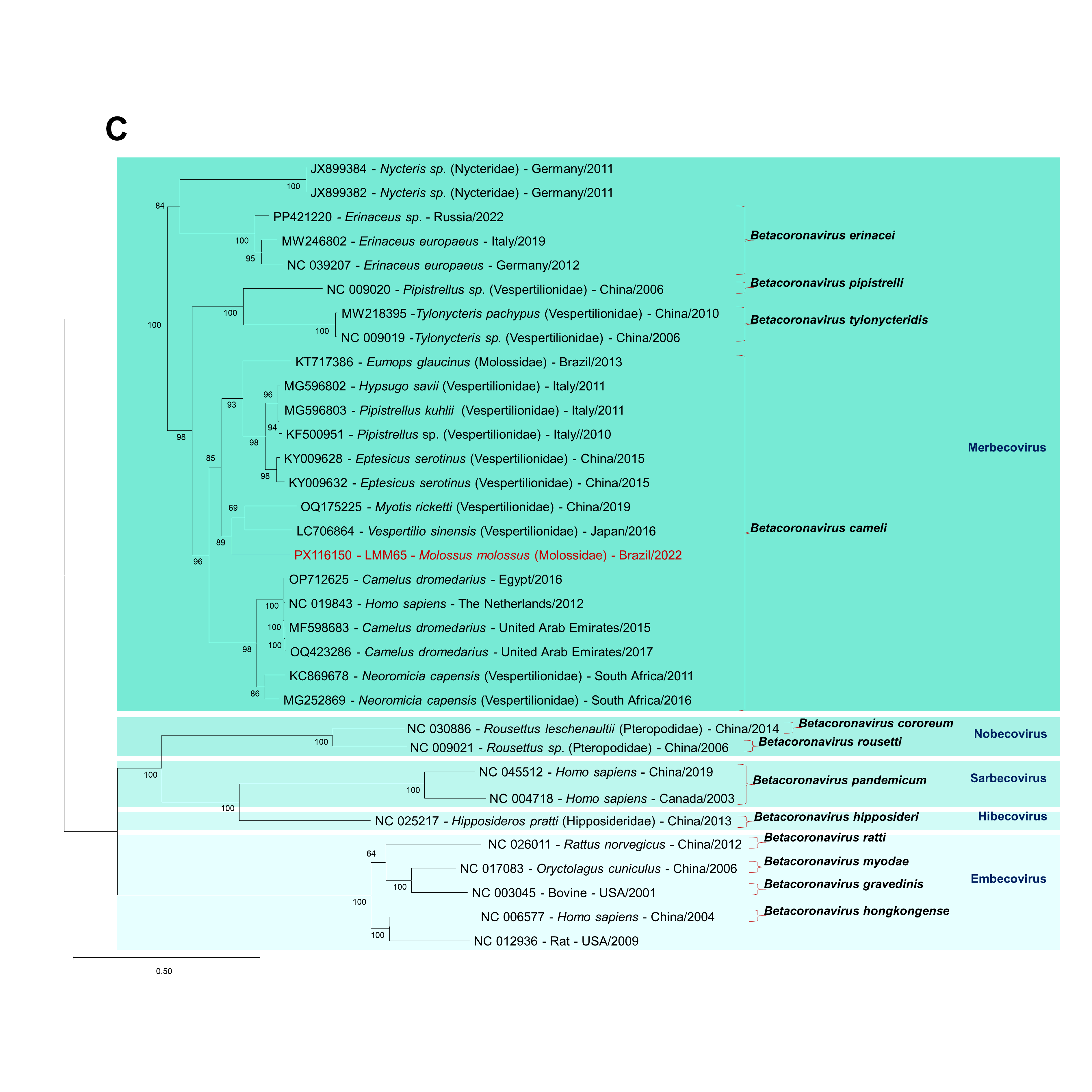

Supplement: Supplementary file 2 — Supplementary Material 1 Figure S1. Nucleotide phylogenetic reconstruction of ORF1ab partial sequences constructed using IQ-TREE software using maximum likelihood inference the GTR+F+I+G4 model in 1,000 bootstrap replicates. High Resolution Image (TIF 1.61 MB) [file 42770_2026_2034_MOESM1_ESM.tif]

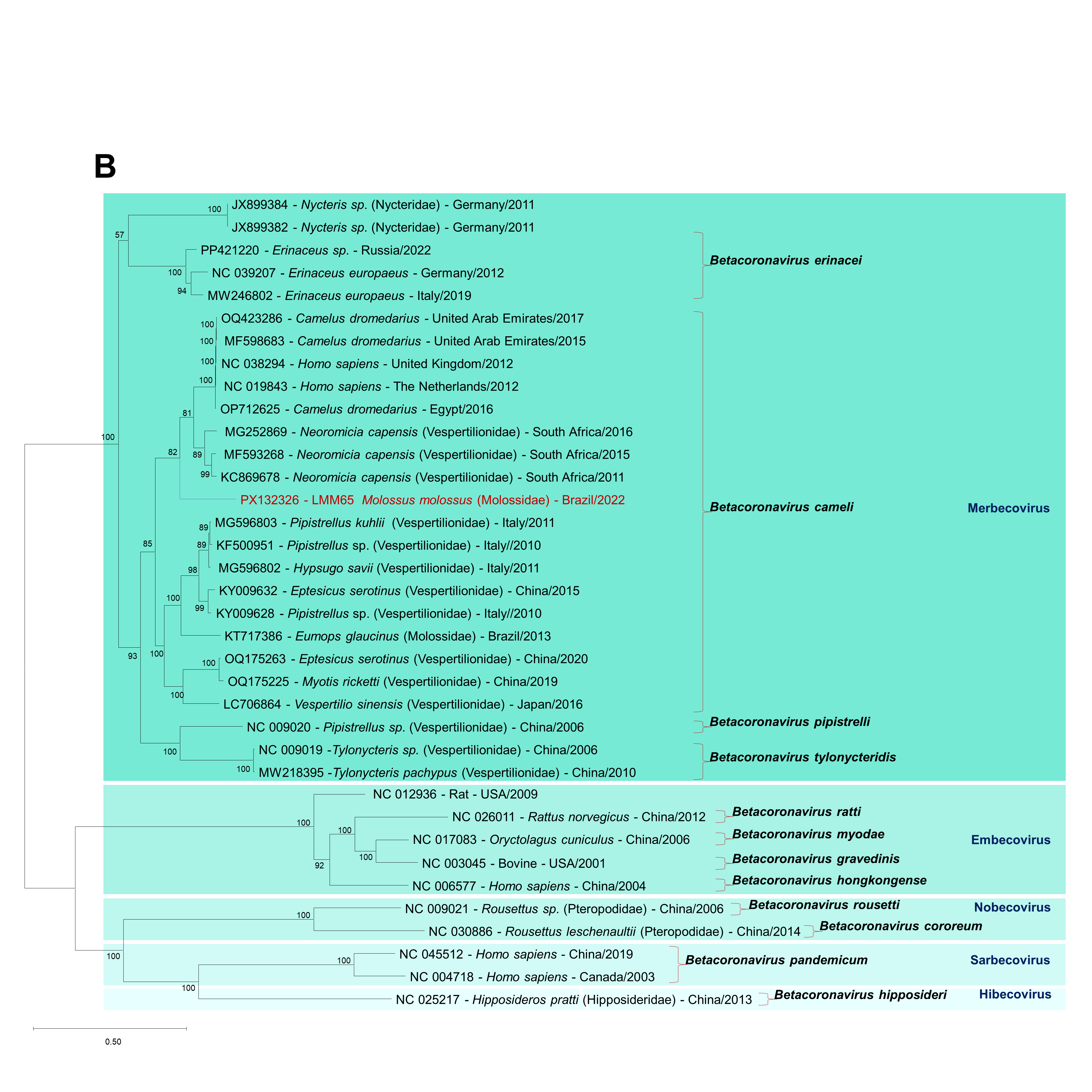

Supplement: Supplementary file 3 — Supplementary figure 2 (PNG 632 KB) [file 42770_2026_2034_Fig4_ESM.png]

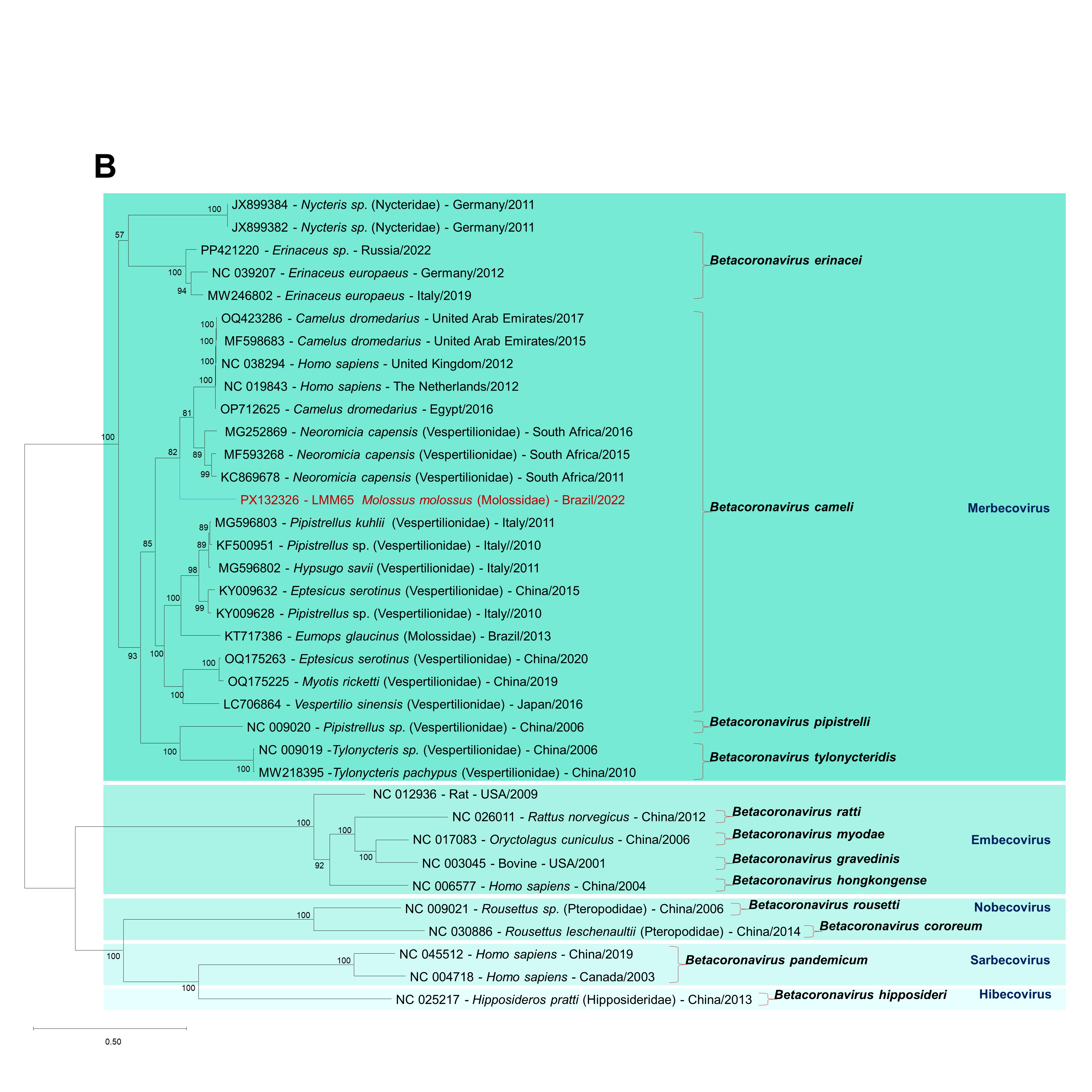

Supplement: Supplementary file 4 — Supplementary Material 2 Figure S2. Nucleotide phylogenetic reconstruction of ORF1ab partial sequences constructed using IQ-TREE software using maximum likelihood inference the GTR+F+I+G4 model in 1,000 bootstrap replicates. High Resolution Image (TIF 1.70 MB) [file 42770_2026_2034_MOESM2_ESM.tif]

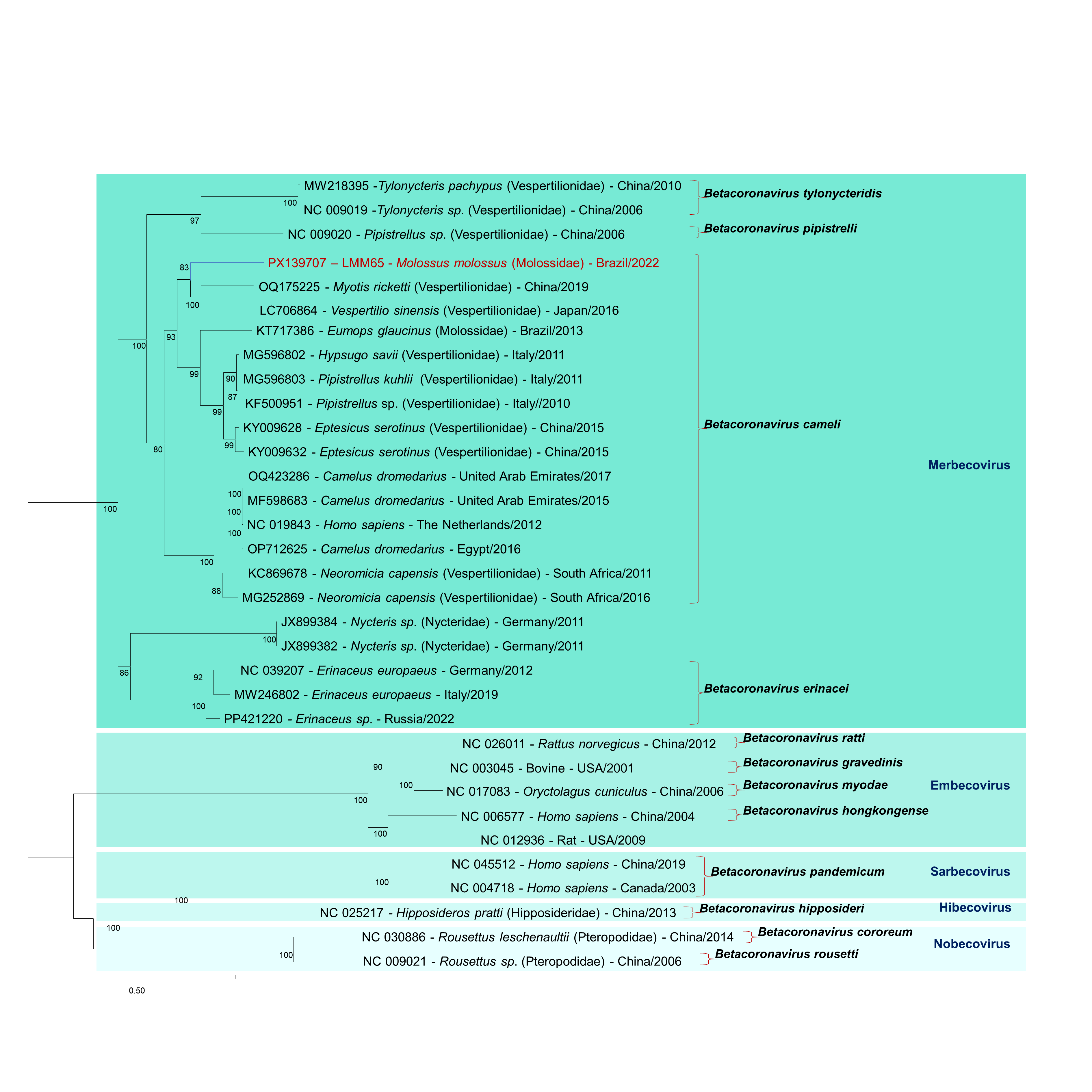

Supplement: Supplementary file 5 — Supplementary figure 3 (PNG 588 KB) [file 42770_2026_2034_Fig5_ESM.png]

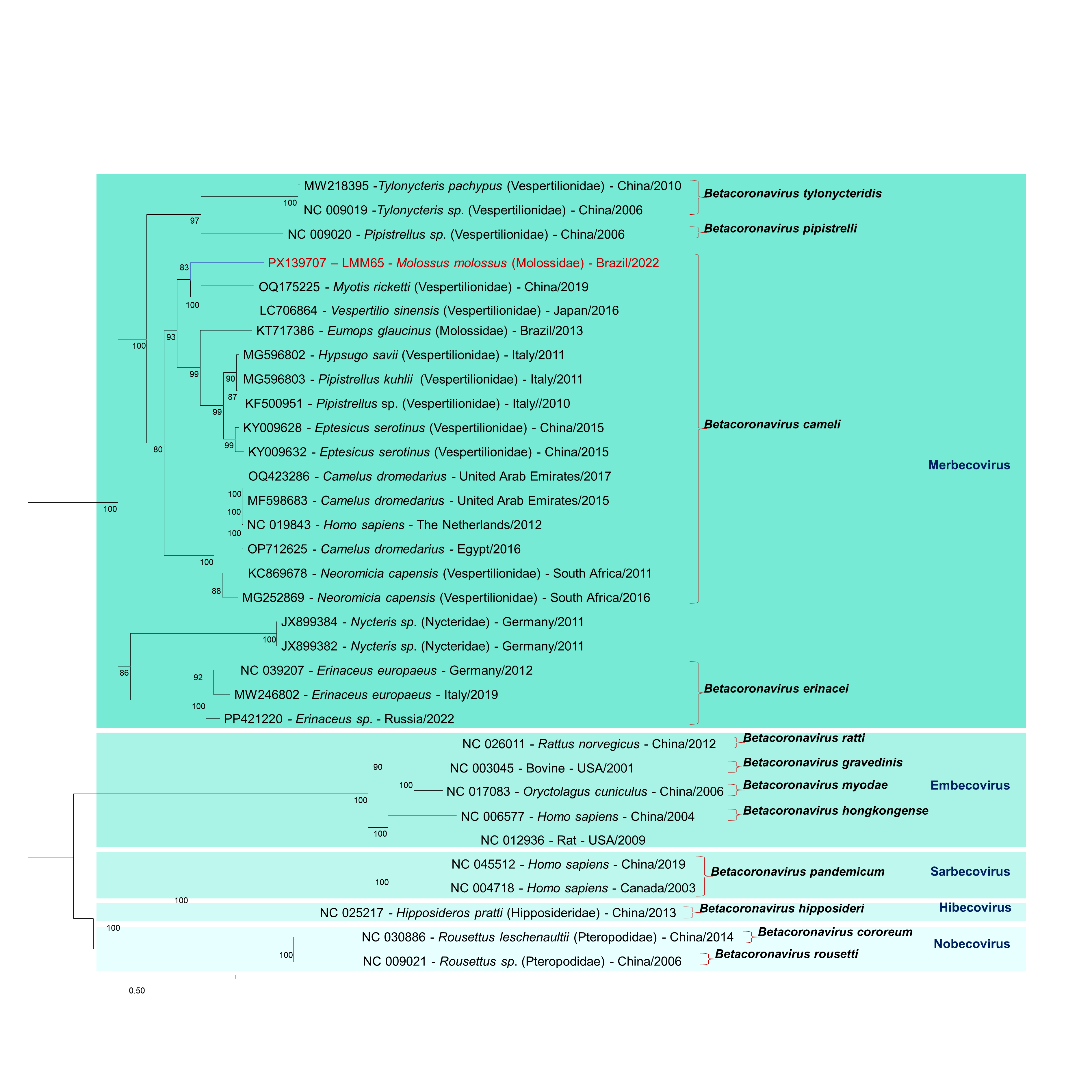

Supplement: Supplementary file 6 — Supplementary Material 3 Figure S3. Nucleotide phylogenetic reconstruction of ORF1ab partial sequences constructed using IQ-TREE software using maximum likelihood inference the GTR+F+I+G4 model in 1,000 bootstrap replicates. High Resolution Image (TIF 1.61 MB) [file 42770_2026_2034_MOESM3_ESM.tif]

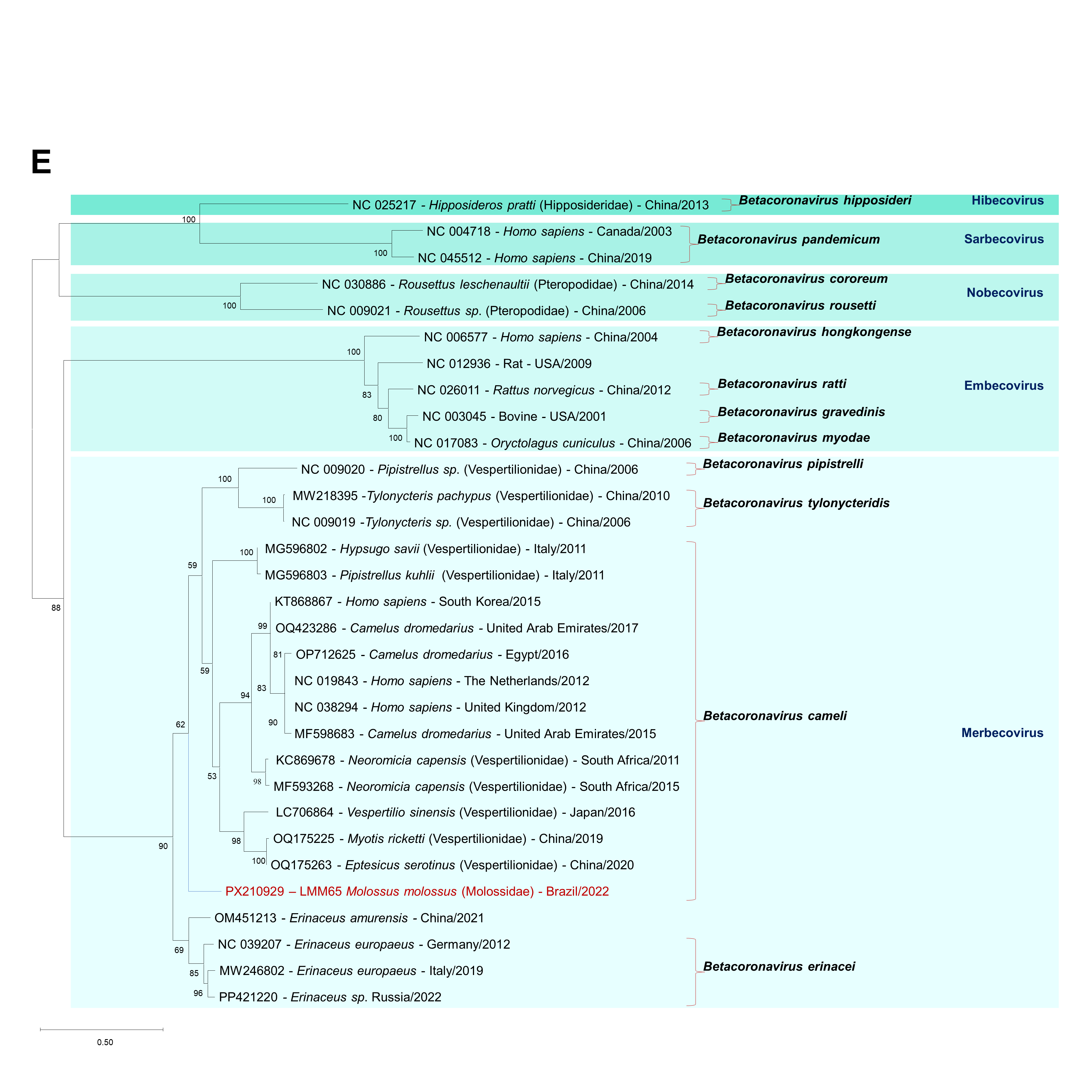

Supplement: Supplementary file 7 — Supplementary figure 4 (PNG 564 KB) [file 42770_2026_2034_Fig6_ESM.png]

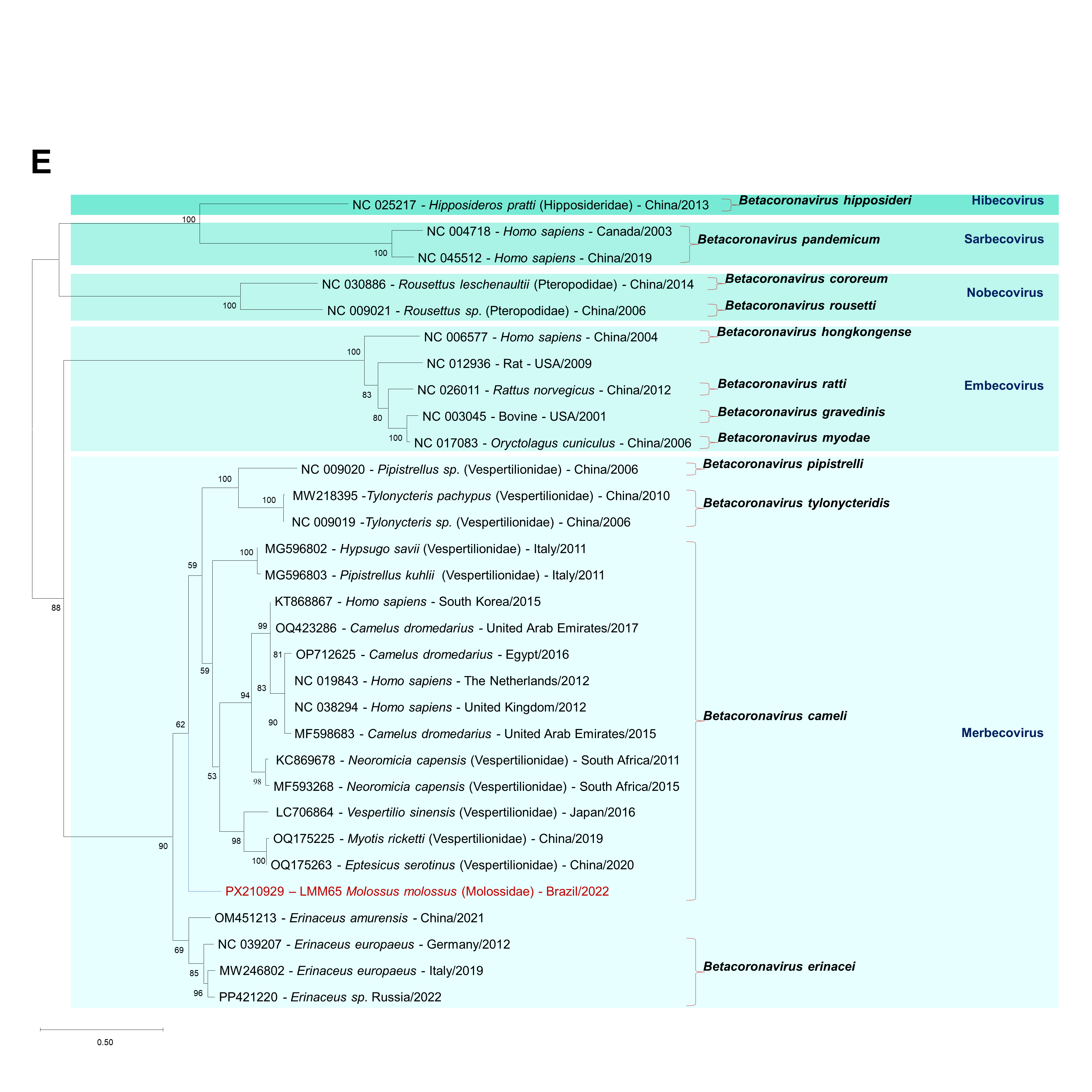

Supplement: Supplementary file 8 — Supplementary Material 4 Figure S4. Nucleotide phylogenetic reconstruction of membrane (M) protein partial sequences constructed using IQ-TREE software using maximum likelihood inference the GTR+F+I+G4 model in 1,000 bootstrap replicates. High Resolution Image (TIF 1.54 MB) [file 42770_2026_2034_MOESM4_ESM.tif]
